# Qualitative exploration of the Medical Examiner role in identifying problems with the quality of patient care

**DOI:** 10.1136/bmjopen-2020-048007

**Published:** 2021-02-05

**Authors:** Rachel O’Hara, Joanne Coster, Steve Goodacre

**Affiliations:** School of Health and Related Research (ScHARR), The University of Sheffield, Sheffield, UK

**Keywords:** qualitative research, quality in health care, clinical governance

## Abstract

**Objective:**

A national system of Medical Examiners (MEs) implemented in England and Wales from April 2019 was intended to ensure that every death receives scrutiny from an independent, senior doctor, resulting in early detection of problems in care. The aim of this study was to increase understanding of how the ME role operates to identify problems related to quality of patient care and to explore the potential for development to maximise learning opportunities.

**Design:**

A qualitative approach involved the use of semi-structured interviews. Data analysis employed a framework approach.

**Setting:**

Study participants were recruited from 11 acute hospitals in England, known to be operating an ME service.

**Participants:**

A purposive sample of 20 MEs and one ME officer.

**Results:**

MEs brought different perspectives to the role based on their medical background. The process for identifying and acting on quality of care concerns was broadly consistent, with a notable consensus regarding the value of speaking to bereaved relatives. Variation was identified within and between services in relation to how core components are carried out and the perceived salience of information, which appeared to reflect individual and service preferences as well as different organisational pathways. ME services required flexibility to accommodate fluctuating demand, but funding arrangements imposed restrictions. The majority of MEs highlighted limited opportunity for formal team contact and a lack of meaningful feedback as limiting scope for development.

**Conclusion:**

Core components of the ME role were being conducted, although individual and systemic variations in practice were identified. The discussion with bereaved relatives is a unique feature of the ME role and was considered highly valuable, both for the organisation and relatives. Further development could consider the impact of the variation identified and address mechanisms for feedback and shared learning.

**Strengths and limitations of this study**

- By providing insight on how this new role has been implemented and is operating, the study can inform further development of Medical Examiner (ME) services.
- Although the number of interviewees reflected a pragmatic decision based on the expected number of services, analysis indicated that data saturation had been achieved.
- Patient and public involvement (PPI) representatives contributed to the design and conduct of this study through input at PPI and advisory group meetings.
- Interviews with the various stakeholders that interface with the MEs could have enhanced the study findings.
- This study has not collected data on the impact of the ME services; further research could address this and also examine the influence of variation and the views of bereaved relatives.

## Background

Serious problems in care resulting in harm to patients and avoidable deaths led to several high-profile investigations and reviews of deaths between 2003 and 2015 in the UK.^1–3^ The introduction of a Medical Examiner (ME) role was recommended to address concerns around death certification in response to the investigation of murders committed by the English general practitioner Harold Shipman^1^ and endorsed in subsequent investigations of avoidable deaths and patient safety as a way of identifying problems in care.^2 3^ The quality of care issues identified by these investigations are not unique to the UK and it has been suggested that other countries could learn from the resulting action, to improve hospital quality and safety governance systems.^4^

Attending doctors are required to notify the local coroner of deaths in specific circumstances (eg, suspected unnatural death) and where the cause of death cannot be established.^5^ The ME role was introduced to provide scrutiny by an independent, senior doctor of all deaths not investigated by a coroner.^6^ The Coroners and Justice Act 2009 provides the legal basis for this new role, but it is not yet a statutory requirement.^7^ The main aims of the ME role are to ensure that the Medical Certificate of Cause of Death (MCCD) is as accurate as possible, that referrals to the coroner are both timely and accurate, and that clinical governance concerns are identified early and referred for more detailed investigation. This is achieved through review of the medical records, discussion with the attending doctor and discussion with the bereaved relatives to communicate the cause of death and identify any concerns.^6 8^ This is different to the US Medical Examiner system, where a more forensic and investigative approach is undertaken for deaths in suspicious or unusual circumstances.^9^ The ME role as outlined here is an approach to mortality review, not currently found outside the UK National Health Service.

In 2016, the regulator of healthcare in England (Care Quality Commission) issued a requirement for all acute hospitals to ‘say something about every death’, to address the finding that learning and improvement were not sufficiently prioritised by some acute hospitals, resulting in missed opportunities to improve care.^10^ For example, decisions to review or investigate deaths relied on staff identifying potential problems in the care provided as an opportunity for learning. Families reported that they were not given information about their relatives’ death or asked whether they had questions or concerns. The National Mortality Case Record Review Programme^11^ was subsequently established using retrospective case record review (RCRR) to investigate potential problems in care for selected deaths.^12^ The Care Quality Commission (CQC) also highlighted the role of families and carers in reviewing quality of care, acknowledging that their experience and insight can provide valuable information.^10^ The requirement for input from bereaved relatives is a unique aspect of the ME approach not found in other mortality review systems.

In 2016, ME services were piloted in six hospitals and an evaluation reported that ME scrutiny plus discussions with bereaved relatives provided a consistent source of high-quality information about the quality of care.^13^ National roll-out of the ME service in England and Wales began in April 2019 followed by the publication of good practice guidelines from the National Medical Examiner in January 2020.^14^ A mandatory e-learning programme provides much of the training needed for new MEs. Initial non-statutory implementation is focused on hospital deaths, with the expectation that this will become a statutory system and expand to cover deaths in other healthcare settings and in the community.^6 8^

There are core components of the role that all ME services are expected to deliver as part of the national programme. On a practical level, each new ME service also needs to work with the existing local clinical governance and mortality review processes, so that ME referrals can feed into existing systems. The aim of this study was to increase understanding of how the ME role operates to identify problems related to the quality of patient care and to explore the potential for development to maximise learning opportunities.

## Methods

### Design

A qualitative approach was adopted to explore how ME services were operating in different hospital sites. Semi-structured interviews were conducted with a purposive sample^15^ of 20 MEs and one MEO (Medical Examiner officer) from 11 acute hospitals in England. The MEO’s role provides support to MEs in managing cases from initial notification through to completion.^8^

### Study participants

A target sample of 20 MEs was a pragmatic decision based on knowledge of the potential number of ME services that would be operating. The sample also aimed to include a proportion of MEs from sites participating in related research comparing ME and RCRR assessments. Study participants were recruited from sites known to be operating an ME service, across a wide range of geographical locations and covering seven of the nine coroner regions in England. Sites included ME services that had been established for several years and those operating for less than a year. Recruitment involved initial contact by email to communicate the study information. Snowball sampling was also employed to identify potential participants. It took longer than anticipated to achieve the target sample as it required waiting for newer services to become established. MEs from 11 of the 16 services contacted volunteered to participate.

Three of the original six pilot services^13^ were included in the study. In four of the 11 services, interviews were conducted with 3–4 MEs to gain a range of perspectives. A fourth ME and an MEO were interviewed to explore specific variations in practice. The MEO was interviewed to examine their involvement in carrying out tasks only undertaken by the MEs in other services. Table 1 shows the number of interview participants per service and length of time the service had been operating when the interviews were conducted. The MEO is not included in the table to avoid identifying individual sites.

**Table 1:** Interview participants—numbers per ME service and time service has been operating

Site	A	B	C	D	E	F	G	H	I	J	K
No of MEs interviewed	4	3	3	1	3	1	1	1	1	1	1
Time service operating (years)	>8	>8	>8	1	1	<1	2	<1	<1	<1	<1

ME, Medical Examiner.

### Data collection

Semi-structured interviews were conducted by telephone (October 2017–August 2019) and lasted 70–90 min. All interviewees had private contact with the researcher and were informed about the study, provided with study information and invited to complete a consent form. All interviews were audio-recorded. Development of the topic guide by the authors was informed by available information on the ME role from the literature and the wider project team. It was piloted within the initial interviews, with only minor subsequent changes following review and discussion. The interview questions focused on understanding how the ME role and services were operating. The interview topics (box 1) and related prompts were the same for all MEs and modified slightly to make them appropriate to exploring similar issues with the MEO (see online supplemental file for interview guide). The semi-structured interview approach ensured that specific topics of interest were addressed, but also allowed the flexibility to explore these or new topics in more detail where appropriate The same researcher (RO) conducted all interviews and is an experienced qualitative healthcare researcher with a PhD in occupational psychology.

10.1136/bmjopen-2020-048007.supp1 Supplementary data

Box 1 **Interview topics**

- Background information—Medical Examiner (ME) and ME Service
- Key components of ME role
- How the role is perceived
- How MEs gather information
- How information is used to make decisions about further investigation
- Links between ME review and retrospective case record review
- Factors that aid or impede the role
- Skills needed for the role
- How the ME role could be developed to improve and maximise learning outcomes
- Lessons for implementation

### Data analysis

Audio-recordings of interviews were transcribed verbatim and checked for accuracy by RO and JC. The data were analysed using a framework approach^15^ supported by NVivo qualitative data analysis software.^16^ The analysis was undertaken by RO and JC. The initial coding framework was aligned with the key interview topics to characterise each of the ME services. Coding was conducted by RO and reviewed by JC. Subsequent iterations of the analysis focused on identifying areas of consistency and variation. Although the number of interviewees reflected a pragmatic decision based on the expected number of services, analysis indicated that data saturation had been achieved in relation to understanding consistency and variation between services.

The interview topic guide, coding framework and emerging findings were discussed with the study team, patient and public involvement representatives and the advisory group.

### Patient and public involvement

Patient and public involvement (PPI) representatives contributed to the design and conduct of this study through input at PPI and advisory group meetings. Preliminary findings were discussed at a local PPI event for the study.

## Results

The results address five features of how the ME role operates to identify problems in care in order to understand the potential for development to maximise learning opportunities. Illustrative interview quotes are presented in table 2.

**Table 2:** Features of how the ME role operates to identify problems in care—illustrative interview quotes

**Who are MEs and what does the role require?**	
Different specialties perceived as beneficial	*Medical examiners, all of them at the moment that I know are consultants. But they are from various different specialities. So there are two of us who are geriatricians, but there are anaesthetists, surgeons, palliative care consultants. So there is a variety of specialties, which is very useful and important actually.* ME10 *My background as a pathologist probably helps because I’ve spent my life, my professional life, reading clinical notes about people who have died. ME18* *We were playing catch up a little bit as pathologists, I think it was very much about formulating the cause of death.(…) governance issues, safeguarding issues are something that has been brought to our awareness as pathologists whereas the clinicians, that’s part of their everyday job so I think that’s enhanced the role.* ME4
MEs should not focus solely on issues linked to cause of death	*End of life care is the biggest single category of problems we’re picking up, which says something about the ill-advisedness on focusing on avoidable deaths because we’re talking about people where death isn’t avoidable but things go really badly wrong with end of life care*. ME8
ME differences may generate inconsistency	*I think the biggest issue that we’ve had to deal with is getting consistency between the different medical examiners*. ME3 *Sometimes we may miss certain things or not consider them particularly relevant because we are not into that speciality particularly. And that’s why I think it would be good to have, you know, quality control with all the MEs*. ME10
The ME role may not suit all doctors	*I think it’s necessary to have experience, some clinical credibility and I think the ability to raise concerns without causing huge drama.(…) But it’s not for everybody, that’s for sure, because I think you’ve got to have the ability to communicate with families in a sensitive manner but not be frightened to tackle difficult clinical governance issues within the organisation*. ME12
**What does the ME review process involve?**	
The process was not a forensic review	*I think we have to be very careful that we are seen as a screening process, trying to identify where there are potential problems, but not trying to sort the problems out*. ME7 *At the end of the day my responsibility here is to identify issues that I believe require further investigation and not to investigate them myself*. ME3
Variation in the order of obtaining information from attending doctors and medical records	*I make a judgement from the notes from what’s gone on, about whether what I think, has happened, and therefore the junior doctors really confirm that. And I use the family as a way to check that what I’ve read in the notes, there’s nothing else gone on that I’ve not been made aware of*. ME20 *The process is in two parts, first of all it’s talking to the certifying doctor or the doctor who's going to refer to the coroner, that is purely a conversation we don’t look at any notes or talk to anybody else at that stage because we didn’t want to delay issuing the death certificate until the end of the medical examiner process the medical examiner then does what we call the screening process*. ME8 *There is a risk I think, that a medical examiner who has already had an interaction with the doctor would look for the confirmatory aspects of that case when they review the records*. ME1
The perceived value of speaking to relatives	*Talking to the family, is the part that you cannot get from the notes*. ME15 *90% of relatives are more than happy, but I think when you do get an unhappy relative, it is helpful to them to speak to someone with a degree of seniority, and it also is a pointer to us, to actually look with a little bit finer toothcomb through the records. [Example of care issue] That sort of thing you wouldn’t necessarily just pick up automatically from the notes.(…) That discussion with the daughter highlighted that there was clearly an issue*. ME6
The perceived value of the service to bereaved relatives	*We did survey families anonymously and asked them to give feedback about that call, it was unanimously universally positive, they liked the idea that somebody independent has reviewed things, they liked the opportunity to get something off their chest, it was an opportunity to have some questions answered, a few felt it was unnecessary because they had no concerns*. ME1 *I do think people appreciate a doctor phoning them, I think they appreciate that time that’s given to them, I think they appreciate the breakdown of medical terminology if needs be.(…) I always explain that I’m an independent doctor(…) and that I haven’t been one of the doctors caring for them.* ME4
Delegation of specific tasks to suitably qualified MEOs	*Rather than having a medical examiner sat in the office all day, every day, what our medical examiners do is they come in for a couple of hours, they review the cases and then they go away. And it’s the MEOs that coordinate all the office functions and have discussions with the doctors and with the family and with the bereaved and with the bereavement office.(…) The contact with the relatives is always done after the medical examiners review.(…) We always feed everything back to the medical examiner, whether it’s discussions with the family or discussions with the doctor.(…) It’s always the medical examiner that makes the ultimate decision, as they have to sign off on the case*. MEO *They’re [MEOs] very experienced, and they will often highlight, things that, need to be reviewed, so they generally look at the records before we do, and say, we’re a bit concerned about X or Y*. ME2
**How do MEs act on potential quality of care problems?**	
Referral via internal pathways when problems are unlikely to have contributed to death, but there appears to be potential learning	*They’re quite often omissions, maybe omissions in recording, maybe omissions in a drug or omissions in undertaking an investigation*. ME7 *Issues with the choice of antibiotics, which might not be the best ones, and there were some issues with the escalation of the patient. Which ultimately didn’t contribute to the cause of death so we didn’t think it was necessary to refer to the coroner, but we felt there was some learning there*. ME17 *Where I don’t think the fluid management has been particularly helpful, there’s been a delay to them getting antibiotics, but I don’t think that delay has contributed to their death. Those sort of things, where I think there’s learning, and process that needs to be improved*. ME20
Referrals for RCRR are also influenced by national and local requirements	*The Trust [hospital] doesn’t influence what the medical examiners look at or do, but(…) we recently discovered that the Trust was an outlier in terms of deaths after myocardial infarction, and there’s one or two other things like that, where the Trust wants to have a particularly close scrutiny of that type of death(…) and for that period all deaths fitting into the category that the Trust’s defined are sent for more detailed case note review*. ME8 *We’ve introduced certain parameters, fractured neck of femur actually is one of them, where all of those cases go forward to the structured review*. ME3
Differences in implicit criteria for referral of concerns to the various internal pathways available	*I would certainly have very low threshold to refer for SJR [RCRR] if there are a family concerns*. ME10 *I also quite frequently pick up things that I haven’t picked up from the notes that the relatives are unhappy about, sometimes that will lead to a structured judgement review, more often it will result in a clinical review*. ME7 *If I am doing a PALS [referral], I would do an SJR [RCRR] as well because obviously there has been a concern raised.* ME15 *It would be a very small number [of relatives] do have questions about quality of care, I can count them on less than one hand I would think, and then you forward them to the PALS service*. ME5
**How are ME services organised?**	
Covering multiple hospitals presented challenges, for example, delayed access to records	*So deaths at the other two sites,(…) it’s probably the area I feel least comfortable about. If we get the notes back within two to three weeks of death, then we will phone up the relatives to talk through with them about the care the patient received.(…) I don’t feel overly comfortable about that because it’s a difficult time for relatives, it’s two or three weeks down the line*. ME6
Electronic records afforded greater flexibility for MEs	*The medical records are electronic and so what I have got into the habit of doing most days, is logging on either in my office or remotely… so I can flick through and review those patient records, either in my office or remotely and make an initial assessment of what the direction of travel looks like*. ME14
Administrative support was beneficial in helping MEs to manage their workload	*MEO’s are essential because they have the heads up on everything really, so when I go to bereavement in the morning, if there are tricky cases, difficult relatives, particularly complex cases they’re on it straight away and they make us aware of that so that when we get the phone call from the junior, we already have an idea of what’s coming our way*. ME4 *They [ME assistant] basically prepare all the notes for us, they make sure that all the details that we need about relatives, contact details, that sort of thing, are all available, they identify the patient episode that resulted in death, they make sure that all the notes are available and appropriately stacked so that we do things in the right order, they liaise with the bereavement office over relatives seeing us*. ME6
Scope for improving the structures for meaningful feedback to ‘close the loop’	*It is really helpful to have feedback though and I think that’s perhaps that one aspect of the system that isn’t as strong yet as it might be, I need to have some kind of quality assurance to know that, am I detecting the right kinds of things and also it’s good to know what learning came out of things and what changes have been made because that can inform future reviews as well*. ME1 *I am pushing it out to a black hole and hoping someone else is taking care of it. The black hole is fairly robust I think and there is monitoring*. ME11 *There is a proper feedback process. There is a central data set from where learning points are taken off and disseminated*. ME9
**How are ME services resourced?**	
Professional accountability perceived to safeguard the independence of MEs	*I do understand the concerns about independence of medical examiners. My only experience in [location] is that those concerns are unfounded because we employ medical examiners who are senior doctors who know that they have a responsibility to the General Medical Council overall, rather than their own Trust [hospital].(…) quite apart from the GMC, medical examiners should have recourse to a National Medical Examiner to say ‘‘this has happened, I think I’m being put under undue pressure’’.* ME8
The flexibility needed to accommodate fluctuating demands and cremation form requirements is challenging	*I struggle with flexibility because of my other jobs*. ME17 *I can try and shift the whole thing around so that I am free for that [ME] work, and that’s how it has to be, especially in winter times, so it’s a matter of me time shifting really*. ME4 *It is quite tricky to say to somebody I will pay you for four hours work but actually I would like you to be available between ten and four*. ME15
Ensuring availability of supporting staff is important to maintain service continuity	*When the MEO isn’t there, it just falls apart*. ME17 *Bereavement care is currently very short- staffed and I am directing more of my energies than I was at the beginning to supporting bereavement care because if they fall over, we’ve got a problem. And it does rather limit the time I’ve got in terms of family follow up. So I’ve switched it for the time being to only following up the families who have requested a cremation*. ME18

### Who are the MEs and what does the role require?

MEs across all services are experienced senior consultant grade doctors or an equivalent career grade (eg, associate surgical specialist trained abroad). Each service employed MEs from a range of medical specialties that were represented among the 20 interviewees: Acute/General Medicine (2); Anaesthesia (1); Elderly care (4); Emergency Medicine (2); Genitourinary Medicine (1); Intensive care (2); Obstetrics & Gynaecology (1); Orthopaedics (1); Palliative care (1); Pathology (4); Respiratory (1). Input from different specialties was regarded as beneficial in bringing different perspectives. Pathologists regarded themselves as more experienced in determining cause of death and dealing with coroners, whereas clinicians tended to view themselves as more focused on the care process. In specialties where death was more likely to be expected (eg, palliative or end-of-life care), the quality of care rather than avoidable mortality was regarded as a particular area of interest. It was considered important that the detection of potential problems in care by MEs should not focus solely on identifying issues implicated in the cause of death. The potential for inconsistency associated with different perspectives was raised.

There was consensus among interviewees that seniority, communication skills and confidence are key for the challenging questions and decisions required of MEs, as well as sensitive conversations with relatives. A number of MEs also commented that the role would not suit everyone who is eligible, thus highlighting the importance of the selection process. All MEs had completed the national online training package developed, which was regarded as a useful generic starting point that needed to be supplemented with more specific local training to familiarise MEs with local systems and internal pathways to refer cases for further review (eg, more detailed mortality review). The extent and nature of additional local training varied, but generally included shadowing and support from ME colleagues, and training events involving internal as well as external stakeholders (eg, coroners, registrars, hospital leads for mortality, safety, governance and bereavement services). Involvement of local stakeholders was regarded as helpful, as was the opportunity to attend national events to meet MEs from other services.

### What does the ME review process involve?

Although the terminology varied (eg, screening, scrutiny), there was agreement that the ME review process was not a forensic review and was intended to identify ‘red flags’ or concerns that prompt referral to the coroner or internal governance systems for more detailed investigation and action as appropriate. ME services were delivering the core components of the ME role as identified in national guidance: screening of clinical notes, including review of other relevant records (eg, diagnostic tests); a discussion with the attending (usually junior) doctor responsible for completing the MCCD; a conversation with the bereaved relatives. At the time the interviews were conducted, the ME services were at different stages of development, reflecting incremental approaches to implementation of this new role and differences in the length of time they had been established. Therefore, not all hospital deaths were included in the full ME review process.

There was some variation between MEs in relation to the order in which they obtained information from attending doctors and medical records. Some of this was for pragmatic reasons (eg, availability of doctors). Specific approaches advocated within the ME service also contributed to variation, for example, one service had a more distinct differentiation between reviewing the cause of death and clinical governance review. MEs also expressed individual preferences about whether they reviewed the medical record or spoke to the attending doctors first.

Speaking to bereaved relatives was highly valued by interviewees across all services and was perceived to be valued by the relatives, who had the cause of death explained and were given the opportunity to ask questions and provide feedback on the care received. A script was used to guide these conversations in some services and was recommended as helpful for newer MEs. Information from relatives rarely changed opinions regarding the cause of death but did help identify potential quality-of-care issues within the hospital as well as the community setting (eg, care homes, ambulance service, primary care). Examination of the deceased was carried out by MEs involved in completion of cremation forms (a mandatory requirement) but was not considered informative in ME decision-making. It was difficult to establish a precise duration for the review process due to differences in the complexity of cases; approximately 30 min was most common and up to a maximum of 60 min to complete cremation forms or for particularly complicated cases.

In one service, a notable difference in the ME review process involved the delegation of specific tasks to MEOs with backgrounds in biomedical science and nursing. MEOs discussed cases with the attending doctors and spoke to the relatives. MEs reviewed the medical record and approved any decisions. Where appropriate, MEs spoke to attending doctors and relatives to explore or respond to specific concerns. Delegation to MEOs was regarded as beneficial in reducing the demand for ME time. In other services, MEOs provided administrative-based support only.

### How do MEs act on potential quality-of-care problems?

When potential quality-of-care problems were identified, these were referred for further review to the coroner and/or internal governance systems. Coroner referrals were required when problems in care may have contributed to the death, whereas internal governance referrals occurred when the identified issues were unlikely to have contributed to the death but there appeared to be potential for organisational learning and improvement (eg, delays in escalation and transition to end-of-life care or omissions in recording). MEs identified variation in how they addressed potential governance concerns in coroner referrals, either simultaneously making a clinical governance referral or waiting for completion of the coroner review.

MEs identified various internal pathways to act on quality-of-care concerns, including clinical governance teams, RCRR, serious incidents, root cause analysis, morbidity and mortality meetings, clinical team review, and the Patient Advice and Liaison Service (PALS). The most consistent route for more detailed scrutiny was RCRR. Referrals for RCRR also included selected deaths mandated for investigation by the Learning from Deaths Programme. Some ME services were also asked by their hospital to refer certain deaths for further review, where they were a source of concern for the organisation (eg, a high number of deaths after myocardial infarction). Sometimes multiple pathways were used to simultaneously prompt further investigation and immediate action.

There appeared to be differences between MEs in the relative salience of information sources and the implicit criteria for referral decisions, which may influence consistency both within and between services. This was most notable in relation to the influence of information from relatives and in some instances appeared related to clinical specialty. Some MEs indicated that they had a ‘low threshold’ for RCRR referral based on relatives’ concerns, whereas others indicated that relatives’ concerns would be less likely to trigger referral for RCRR and tended to be referred to PALS. New referral pathways had been introduced to act on issues identified by MEs but considered not to merit RCRR, for example, direct feedback to clinical teams regarding concerns, as well as compliments from relatives. Local systems for documenting information also varied, though some services had learnt from other settings and adopted similar approaches.

### How are ME services organised?

The need for ME services to integrate with existing administrative and clinical governance systems within each setting was identified as a source of variation. Ten of the services comprised a team of MEs working for a fixed number of hours or professional activities time each week. The majority of MEs also worked as doctors within their respective hospitals, except for one who was employed for ME-related work only. MEs avoided reviewing cases where they had clinical input. The numbers of MEs per service and number of hours worked varied, generally influenced by the number of deaths. Lead MEs were identified in a number of services, which involved a more strategic focus on the development of the service. None of the services operated out-of-hours except for relatively informal arrangements to expedite release of the body for religious or cultural reasons. Consequently, the ME caseload tended to be higher on Mondays.

Some services covered multiple hospitals and ensuring ME cover across different sites presented challenges, particularly where MEs could not be recruited on site. In one service, concern was raised about delays in accessing paper records where they had to come from another site. This impacted on the timeliness of clinical governance review and conversations with relatives. MCCDs could be completed without delay by attending doctors at the other site. Most of the hospitals used paper records but where electronic records were available they afforded greater flexibility over where and when MEs could review records.

The physical environment was identified as important, as was proximity to MEOs, administrative assistants and bereavement office staff. Administrative support was regarded as beneficial in helping MEs to manage their workload as sourcing and prioritising the notes, and arranging conversations with attending doctors and relatives, was often reported as time consuming and logistically challenging.

The way in which ME services were organised appeared to restrict opportunities for formal team communication, although some had developed approaches to facilitate more direct communication (eg, team meetings, social messaging platform). Feedback and learning were considered particular areas of weakness in most of the ME services. Specifically, the lack of structures for providing meaningful feedback to ‘close the loop’ on problems in care and clinical governance issues identified by MEs, and for sharing learning.

Service guidance and oversight were recommended to improve consistency across MEs but caution was also advised regarding the extent of standardisation, to avoid reducing the process to a ‘tick box exercise’.

### How are ME services resourced?

The majority of MEs were funded via payments for completing cremation related tasks and paperwork, which in most services reimbursed MEs for their input. Other funding included limited Department of Health support for pilot services and from the hospitals. One service was delivered by a sole ME operating in a voluntary capacity pending the appointment of staff for a fully resourced service.

Cremation fees placed constraints on service resources by restricting the ability of MEs to hand over unfinished cases. The same doctor had to complete all components of the ME role plus examine the deceased to meet cremation form requirements. Similarly, these requirements prevented the delegation of ME activities to MEOs as they had to be carried out by a doctor. Variation in funding was identified within services. For example, where there was no ME on-site, the reviews of deaths were funded by the hospital as MEs were unable to meet the cremation form requirement to examine the deceased. Employment by the hospital and funding of ME services by the hospitals in which they are located was not in itself regarded as compromising the independence of MEs. Some MEs referred to their duty of candour as doctors and professional accountability as safeguarding the independence of their practice.

A degree of flexibility in ME availability was identified as necessary to accommodate both fluctuating demands and cremation form requirements. The demands of consultant rotas and role requirements, particularly in some specialties, made this challenging. The availability of staff in supporting roles across service office hours provided continuity and their ongoing availability appeared to be as important as MEs in ensuring continuity of service. Examples were identified in two services where shortages of MEOs and bereavement office staff restricted ME service delivery.

## Discussion

### Summary of findings

MEs bring different perspectives to the role based on their medical background. The process for identifying and acting on potential quality of care concerns was broadly consistent, with notable consensus regarding the value of speaking to bereaved relatives. Variation was identified within and between services in relation to how core components are carried out and the perceived salience of information, which appeared to reflect individual and service preferences as well as different organisational pathways. Resourcing ME services required flexibility to accommodate fluctuating demand but funding via cremation fees placed constraints on services. The majority of MEs highlighted limited opportunity for formal team contact and a lack of meaningful feedback on referrals as limiting scope for development.

### Findings in the context of relevant research

The ME role is relatively new and there does not appear to be a similar approach to learning from deaths outside the UK, therefore relevant research is limited. Research examining review systems intended to be timelier than RCRR by seeking immediate feedback from front-line staff found that this approach identified information on system-level issues not in the patient record.^17–20^ MEs similarly recounted instances where attending doctors provided additional detail, but this did not appear to make a significant contribution to identifying quality-of-care concerns. Analysis of ME reviews in one of the pilot services identified clinical governance issues in 4% of cases that attending doctors were unaware of, including system failures highlighted by bereaved relatives.^6^ This is consistent with ME accounts of the value of feedback from relatives and represents a strength of this approach.

The ME role as carried out appeared generally consistent with the key requirements in relevant guidance.^14^ However, there appeared to be differences between MEs in their implicit criteria for decision-making. Reviewer variation has been identified in other studies, whereby inconsistency develops over time and as the number of reviewers increases; attention to ongoing training was recommended.^18^ The CQC similarly recommends protected time for training and support to reduce inconsistency in how hospitals review and investigate deaths.^10^ Team contact and feedback could also address this issue.

The implementation of the ME role has involved integration with existing processes and systems within each setting, which has contributed to variations between services. Systemic influences on variation have also been identified in the United States ME role, where death investigation meets basic requirements across various states but considerable variation in practice exists due to differences in local requirements, funding, staffing, practice preferences and practice patterns.^9^ Rather than seeking to ensure national consistency, it has been suggested that developing and implementing new systems to deliver consistency in core components of the service, while recognising the need for pragmatism and local creativity, is preferable for sustainable and meaningful service improvement.^21^

### Implications

The ME role has the potential to make a significant contribution to improving the quality of patient care by identifying potential problems and referring them for further investigation and action. As the role becomes embedded within all hospitals, there is scope for sharing lessons on a wider scale. It also offers the potential to influence patient safety culture by supporting organisational improvement and learning.^18^ The consistency and variation identified within and between ME services encompassed multiple influences at the level of the individual MEs, the ME services, the wider organisational systems and structures, and external policy and guidance. Fulop and Ramsay outline how organisational processes and leadership in acute hospitals can bridge the gap between these different levels of influence to improve the quality of healthcare.^22^ For ME services, this would include well-supported strategies and systems to ensure concerns raised by MEs are investigated, findings acted on and learning shared. However, the CQC found a lack of consistent systems in place to ensure that recommendations from investigations were acted on and learning shared, beyond single settings.^10^

### Strengths and limitations

This new approach to learning from deaths seems easily transferable to other healthcare settings and the insights provided here can inform implementation to maximise learning opportunities. The services in this study are drawn from a small proportion of hospitals in England and may represent those more proactive in implementing this new role. The findings would have been strengthened by having more than one interviewee in all of the services. Interviews with the various stakeholders that interface with the MEs could have enhanced the study findings by providing insight into what happens to cases referred for further investigation. The interview approach is subject to the limitations of individual recall and observation of practice could have provided more reliable information, but that was not feasible within the study resources. This study has not collected data on the impact of the ME services; further research could address this and examine the influence of variation and the views of bereaved relatives. The data collection was completed prior to the COVID-19 pandemic, and further research could explore how services have responded to this crisis and any lessons that can be learnt.

## Conclusion

The ME role represents a novel approach to identifying quality-of-care problems through mortality review. Core components of the ME role were being conducted, although individual and systemic variations in practice were identified. All MEs reviewed the medical record, with differences in whether this happened before or after speaking to attending doctors. The discussion with bereaved relatives is a unique feature of the ME service and was considered by MEs as highly valuable, both for the organisation and relatives. The potential for variation in referrals for further investigation relates to differences in organisational pathways and individual decision criteria. Further development could consider the impact of the variation identified and address mechanisms for feedback and shared learning.

## Supplementary Material

Author's manuscript

## References

[1] Smith DJ . The Shipman inquiry, third report: death certification and the investigation of deaths by Coroners. London, 2003. Available: https://www.gov.uk/government/uploads/system/uploads/attachment_data/file/273227/5854.pdf [Accessed 26 Jan 2018].

[2] Francis R . Report of the Mid Staffordshire NHS Foundation Trust Public Inquiry, 2013. Available: www.midstaffspublicinquiry.com [Accessed 26 Jan 2018].

[3] Kirkup B . The report of the Morecambe Bay investigation, 2015. Available: https://www.gov.uk/government/publications/morecambe-bay-investigation-report [Accessed 26 Jan 2018].

[4] Shaw C , Kutryba B , Crisp H , et al. Do European hospitals have quality and safety governance systems and structures in place? Qual Saf Health Care 2009;18 Suppl 1:i51–6. 10.1136/qshc.2008.029306 19188462 PMC2629926

[5] Legislation.gov.uk. The notification of deaths regulations, 2019. Available: https://www.legislation.gov.uk/uksi/2019/1112/made [Accessed 26 Oct 2020].

[6] Fletcher A , Coster J , Goodacre S . Impact of the new Medical Examiner role on patient safety. BMJ 2018;363:k5166. 10.1136/bmj.k5166 30552229

[7] Legislation.gov.uk. Coroners and Justice Act 2009, 2009. Available: http://www.legislation.gov.uk/ukpga/2009/25/contents [Accessed 26 Jan 2018].

[8] NHS Improvement. The National Medical Examiner System. Available: https://improvement.nhs.uk/resources/establishing-medical-examiner-system-nhs/ [Accessed 17 Apr 2020].

[9] Hanzlick RL . A perspective on medicolegal death investigation in the United States: 2013. Acad Forensic Pathol 2014;4:2–9. 10.23907/2014.001

[10] Care Quality Commission. Learning, candour and accountability: a review of the way NHS trusts review and investigate the deaths of patients in England, 2016. Available: https://www.cqc.org.uk/sites/default/files/20161213-learning-candour-accountability-full-report.pdf [Accessed 26 Jan 2018].

[11] Royal College of Physicians. National mortality case record review programme. Available: https://www.rcplondon.ac.uk/projects/national-mortality-case-record-review-programme [Accessed 19 Mar 2020].

[12] NHS. Learning from deaths in the NHS. Available: https://improvement.nhs.uk/resources/learning-deaths-nhs/ [Accessed 19 Mar 2020].

[13] Furness PN , Fletcher AK , Shepherd NA . Reforming death certification: introducing scrutiny by Medical Examiners. Lessons from the pilots of the reforms set out in the Coroners and Justice Act 2009. Department of Health, 2016. Available: https://assets.publishing.service.gov.uk/government/uploads/system/uploads/attachment_data/file/521226/Death_certificate_reforms_pilots_-_report_A.pdf [Accessed 19 Mar 2020].

[14] NHS. Implementing the medical examiner system: national medical examiner’s good practice guidelines. NHS England and NHS Improvement, 2020. Available: https://improvement.nhs.uk/documents/6398/National_Medical_Examiner_-_good_practice_guidelines.pdf [Accessed 19 Mar 2020].

[15] Ritchie J , Lewis J , Nicholls CM . Qualitative research practice: a guide for social science students and researchers. 2 edn. London: Sage, 2013.

[16] NVivo. NVivo qualitative data analysis software; version 12. QSR International Pty Ltd, 2018.

[17] Kobewka DM , van Walraven C , Turnbull J , et al. Quality gaps identified through mortality review. BMJ Qual Saf 2017;26:141–9. 10.1136/bmjqs-2015-004735 PMC5284344 26856617

[18] Huddleston JM , Diedrich DA , Kinsey GC , et al. Learning from every death. J Patient Saf 2014;10:6–12. 10.1097/PTS.0000000000000053 24553440

[19] Mendu ML , Lu Y , Petersen A , et al. Reflections on implementing a hospital-wide provider-based electronic inpatient mortality review system: lessons learnt. BMJ Qual Saf 2020;29:304–12. 10.1136/bmjqs-2019-009864 31649164

[20] Gupta M , Fuchs B , Cutilli C , et al. Preventable mortality: does the perspective matter when determining preventability? J Surg Res 2013;184:54–60. 10.1016/j.jss.2013.05.069 23773717

[21] Black N . New era for health services will focus on systems and creativity—an essay by Nick Black. BMJ 2018;362:k2605. 10.1136/bmj.k2605

[22] Fulop NJ , Ramsay AIG . How organisations contribute to improving the quality of healthcare. BMJ 2019;365:l1773. 10.1136/bmj.l1773 31048322 PMC6495298

