# Supplementary material for: Qualitative exploration of the Medical Examiner role in identifying problems with the quality of patient care
Source: BMJ Open. 2021 Feb 5;11(2):e048007. doi: 10.1136/bmjopen-2020-048007 (PMC7925852; doi:10.1136/bmjopen-2020-048007)
Supplement: Supplementary data [file bmjopen-2020-048007supp001.pdf]

Safety for Patients through Quality Review (SPQR): Interview topic guide

Study/researcher introduction

We are trying to understand more about the role of the Medical Examiner. In particular, what are the key components of the role and how the role is perceived, to find out about factors that aid or impede the role. We are interested in the practical aspects of the role such as how Medical Examiners gather and use information to make assessments and how the role could be developed further. We will also explore what factors affect decisions, for example local organisational factors or discussions with relatives.

Introductory questions

- How long have you been in post as a medical examiner?
- How were you recruited to the role?
- What was your background prior to this? Do you hold any other roles currently?
- Can you tell me about any training that you've had, specifically for your medical examiner post?
- How many medical examiners are in post at your Trust?
- How many cases do you review per week, on average?

The Medical Examiner role

1. What do you see as the main purpose of the ME role?
2. What are the key components/tasks in your ME role?
3. What factors do you find helpful in undertaking your ME role?
4. Are there any factors that you find unhelpful in undertaking your ME role?

Case review process

5. Can you talk me through the process of what happens when you are reviewing a case?
6. How do you make your decisions about which cases require further investigation or no further investigation?
7. What are your views about the value of discussions with relatives in informing your judgement about a case?
8. What skills are needed to undertake the role effectively?

ME and Retrospective Case Record [RCRR] review

9. What links does your ME case review team have with the RCRR team

Further development

10. How do you see the ME role developing?
11. How can learning opportunities from ME and RCRR be maximised?
12. What advice would you give on implementing the ME role?
